# Probing the second dehydrogenation step in ammonia-borane dehydrocoupling: characterization and reactivity of the key intermediate, B-(cyclotriborazanyl)amine-borane†
†Electronic supplementary information (ESI) available: Experimental procedures, X-ray and NMR data. CCDC 1022830 and 1022831. For ESI and crystallographic data in CIF or other electronic format see DOI: 10.1039/c4sc02710h



**DOI:** 10.1039/c4sc02710h

**Published:** 2014-10-30

**Authors:** Hassan A. Kalviri, Felix Gärtner, Gang Ye, Ilia Korobkov, R. Tom Baker

**Affiliations:** a Department of Chemistry and Centre for Catalysis Research and Innovation (CCRI) , University of Ottawa , 10 Marie Curie , Ottawa , Ontario K1N 6N5 , Canada . Email: rbaker@uottawa.ca ; Fax: +1 613 5625613 ; Tel: +1 613 5625698; b Leibniz-Institut für Katalyse (LIKAT) , Albert-Einstein Straβe 29a , 18059 Rostock , Germany

## Abstract

While thermolysis of ammonia-borane (AB) affords a mixture of aminoborane- and iminoborane oligomers, the most selective metal-based catalysts afford exclusively cyclic iminoborane trimer (borazine) and its B–N cross-linked oligomers (polyborazylene). This catalysed dehydrogenation sequence proceeds through a branched cyclic aminoborane oligomer assigned previously as trimeric B-(cyclodiborazanyl)amine-borane (BCDB). Herein we utilize multinuclear NMR spectroscopy and X-ray crystallography to show instead that this key intermediate is actually *tetrameric* B-(cyclotriborazanyl)amine-borane (BCTB) and a method is presented for its selective synthesis from AB. The reactivity of BCTB upon thermal treatment as well as catalytic dehydrogenation is studied and discussed with regard to facilitating the second dehydrogenation step in AB dehydrocoupling.

## Abstract 2

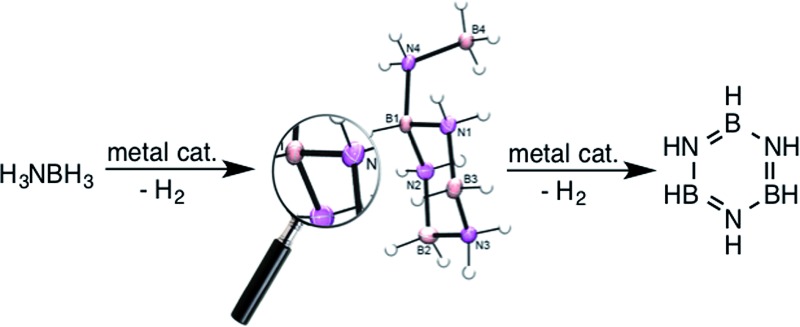
The key intermediate in metal-catalyzed dehydrogenation of ammonia-borane to borazine is shown to be the BN analog of ethylcyclohexane.

## Introduction

The development of clean and efficient technologies for the production, storage and utilization of hydrogen is prerequisite for establishing a hydrogen economy.^1^ For hydrogen storage,^2^ chemical hydrides such as methanol,^3^ formic acid,^4^ carbazoles,^5^ hydrazine^6^ and especially ammonia-borane^7^ are promising sources that could serve as ‘drop-in’ liquid fuels with the existing fuel delivery infrastructure.

**Scheme 1:**
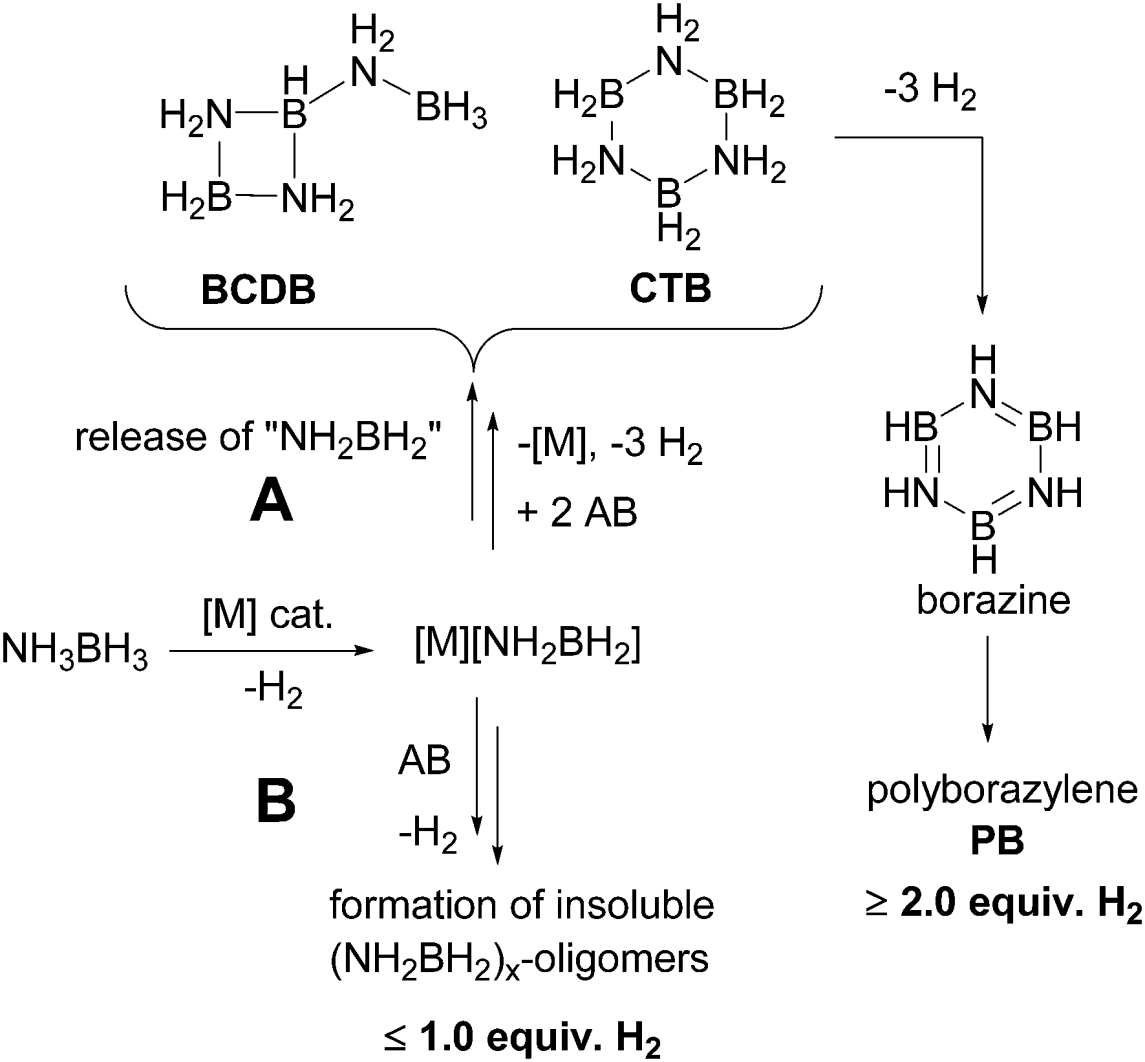
Proposed mechanisms for the metal catalyzed dehydrogenation of ammonia-borane.

**Fig. 1:**
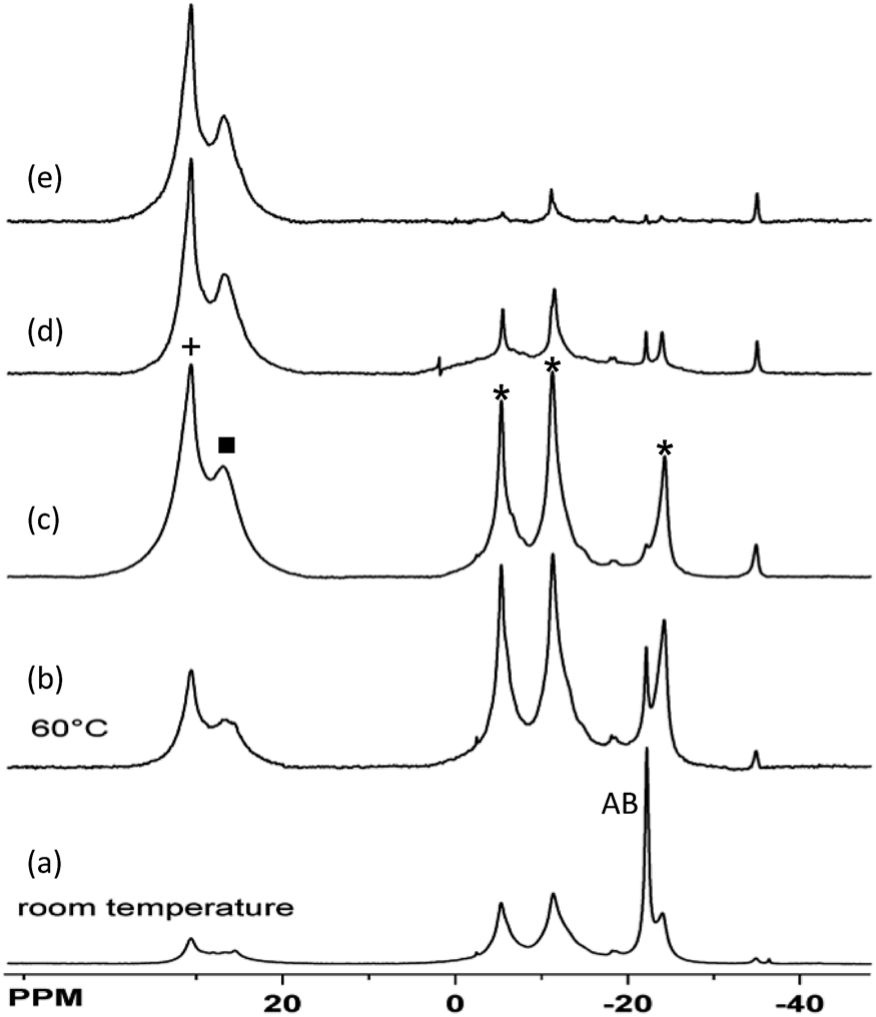
^11^B{^1^H} NMR spectra showing Ni(NHC)_2_-catalyzed conversion of AB to **3** (*) [(a): after 12 h at 20 °C] and of **3** to borazine (+) and polyborazylene (■) [(b)–(e) after heating at 60 °C in the NMR probe (10 min intervals; NHC = 1,3,4-triphenyl-4,5-dihydro-1*H*-1,2,4-triazol-5-ylidene). Minor resonances in top spectrum are due to CTB (–12 ppm) and NHC-borane (–34 ppm).

Ammonia-borane (AB, NH_3_BH_3_) is an especially attractive hydrogen storage material due to its high gravimetric hydrogen content of 194 g H_2_ per kg (19.6 wt% H_2_). Since the molecule contains both hydridic as well as protic hydrogens, hydrogen release can be realized under mild conditions by various means such as thermal treatment,^8^ acid-^9^ or base^10^ catalysis, or metal-based catalysis.^11,12^ A number of AB dehydrocoupling mechanisms have been elucidated^8,9,10,11g–k,13^ and metal-based catalysis has been shown to offer the best combination of selectivity and H_2_ release rate. While many of the very active catalysts afford linear polyaminoborane and a single equivalent of hydrogen,^11b,d,e,13b,14^ the most selective catalysts generate >2 equiv. H_2_ with concomitant formation of cyclic iminoborane trimer (borazine) and its BN-cross-linked oligomers (polyborazylene; Scheme 1).^11c,i,k–m,q,r^ Mechanistic studies of AB dehydrocoupling in ethereal solutions (kinetics, isotope labelling and intermediate trapping) point to the importance of reactive aminoborane, NH_2_BH_2_, shown previously to oligomerize above –150 °C.^15^ It has been suggested that the very active catalysts can retain aminoborane in the first or second coordination sphere and undergo a coordination polymerization-type mechanism (Scheme 1, path B).^13a,16^ In contrast, the selective catalysts expel aminoborane into solution where it undergoes oligomerization to cyclotriborazane (**1**, CTB) and a key branched, cyclic aminoborane intermediate, that we assigned previously as trimeric B-(cyclodiborazanyl)amine-borane (**2**, BCDB). These intermediates then undergo further catalyzed dehydrogenation to the observed borazine and polyborazylene products (Scheme 1, path A and Fig. 1).

In spite of the successful design and development of AB dehydrogenation catalysts, their application is hampered by formation of volatile borazine (bp 51 °C) which contaminates the H_2_ stream and poisons the fuel cell catalyst.^17^ More work is thus needed on understanding and optimizing the second and third H_2_ release steps and in particular on the reactivity of the key cyclic aminoborane intermediate. In this work we describe the first practical synthesis of this intermediate and show unequivocally that it is, in fact, a branched, cyclic aminoborane *tetramer*, B-(cyclotriborazanyl)amine-borane (**3**, BCTB). Reactivity studies demonstrate why thermolysis of **3** yields different products than those derived from its catalytic dehydrogenation.

## Results

### Synthesis and molecular structure of the aminoborane selective oligomerization product

**Fig. 2:**
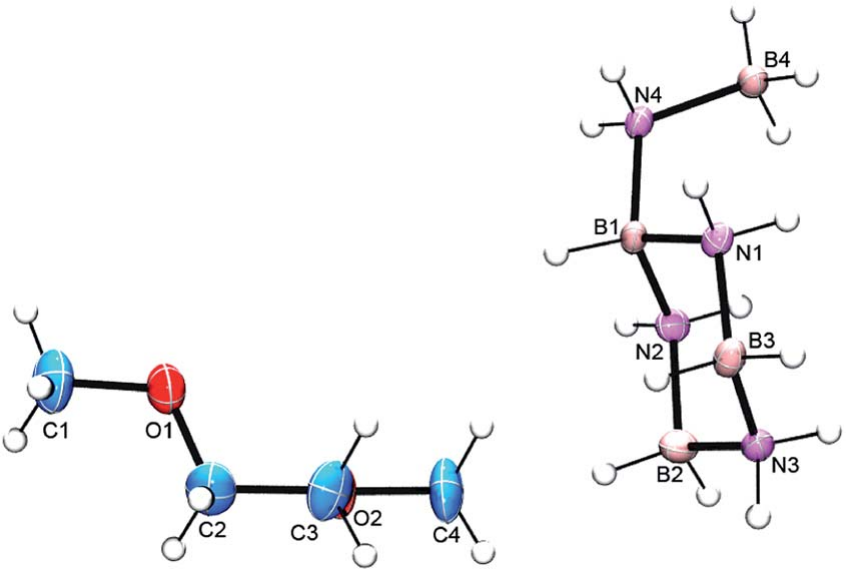
ORTEP view of BCTB (**3**) co-crystallized with glyme. The thermal ellipsoids are drawn at the 50% probability level.

Initial attempts to isolate the key cyclic aminoborane intermediate from AB solution thermolysis at 80 °C, or catalyzed AB dehydrogenation using 10 mol% FeH(CH_2_PMe_2_)(PMe_3_)_3_ (ref. 18) or Ni *N*-heterocyclic carbene precursors^11c,13a^ at room temperature over several days invariably produced impure samples in low yields (ESI,† p. 3). In contrast, reaction of AB with 5 mol% of Schwartz' reagent, Cp_2_ZrHCl (**4**), at 50 °C afforded much purer samples in fair to good yields (ESI,† p. 4). As reported previously,^13*a*^ the ^11^B NMR spectrum of the product consists of doublet, triplet and quartet resonances due to the BH, BH_2_ and BH_3_ groups (Fig. S4†). In previous samples the intensity of the BH_2_ resonance was often seen to be greater than that of the other two, presumed to be due to differing amounts of the aminoborane cyclic trimer, CTB (**1**). After sublimation (80 °C, 2 × 10^–6^ torr for 24 h), however, the desired product was contaminated mostly by a small amount of AB. Although the ^1^H NMR spectrum confirmed just a trace amount of CTB impurity, the BH_2_ intensity in the ^11^B NMR spectrum was still approximately twice that of the other two resonances (Fig. S5 and S6†). Turning to single crystal X-ray crystallography, crystals from the sublimed product were grown *via* two different methods and obtained as a glyme solvate and a crown ether adduct.^19^ Both structures showed the intermediate to be the branched, cyclic aminoborane *tetramer*, B-(cyclotriborazanyl)-amine-borane (**3**, BCTB). (Fig. 2 and S7†). As expected, the bond distances and angles within the 6-membered ring (B3–N3 = 1.572(2) Å, B3–N3–B2 = 116.8(1) ° and N1–B3–N3 = 107.1(1) °) are similar to those in CTB (B–N_avg_ = 1.574(2) Å, B–N–B_avg_ = 115.6(1) ° and N–B–N_avg_ = 107.0(1) °)^20^ and the B4–N4 distance (1.588(2) Å) is similar to analogous bonds in other amine-boranes such as propylamine–borane (1.593(3) Å).^21^ Both structures feature extensive dihydrogen bonding as well as conventional N–H–O hydrogen bonds (Fig. S7 and S8 and Table S6†).

### Proton and nitrogen NMR spectra of BCTB, **3**

**Fig. 3:**
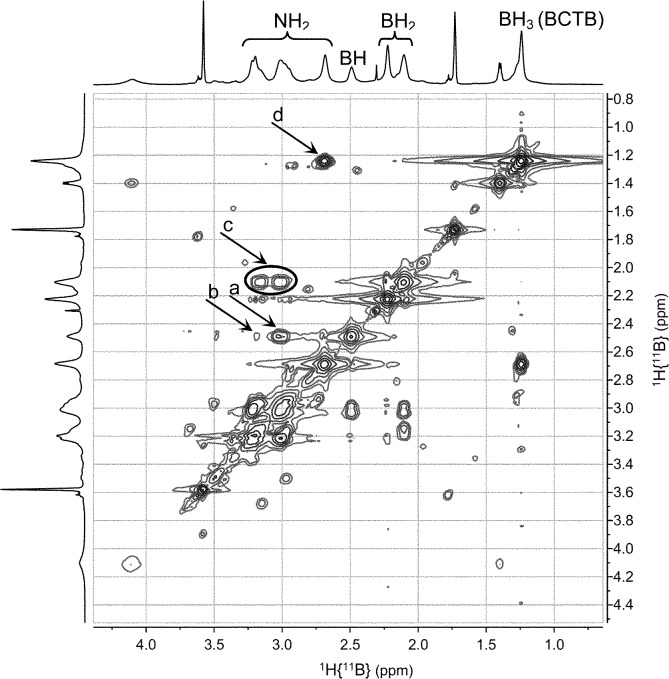
2D-NMR (^1^H–^1^H COSY) (THF-d_8_) of BCTB at –15 °C.

**Fig. 4:**
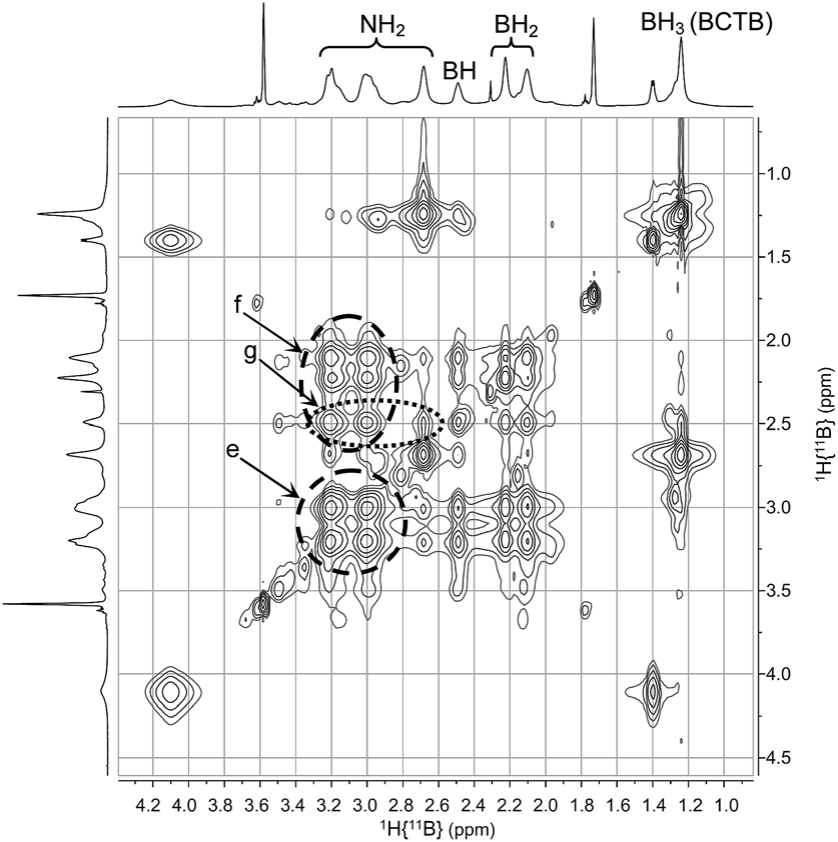
2D-NMR (^1^H–^1^H TOCSY) (THF-d_8_) of BCTB at –15 °C.

A series of multinuclear NMR studies confirmed the structure of BCTB in solution. In the ^11^B decoupled ^1^H/^1^H COSY NMR spectrum at –15 °C (Fig. 3), the B*H* resonance at *δ* 2.5 ppm is strongly correlated with the N*H* at *δ* 3.0 (a in Fig. 3). A weaker correlation is observed with the other N*H* at *δ* 3.2 (b in Fig. 3), thus confirming the chair configuration of the BCTB molecule at low temperature (*i.e.*, strong axial–axial correlations). Moreover, two resonances are observed for B*H*_2_ groups at *δ* 2.1 and 2.2 (c in Fig. 3) with integrations close to 1 : 1 (Fig. S6†). These two peaks can be assigned to the axial and equatorial B*H*_2_ groups in BCTB. Thus at low temperature separate correlations are observed with axial and equatorial protons of N*H*_2_ groups in the ring. On the other hand, the *exo*-N*H*_2_ group (*δ* 2.7) shows a strong correlation with the B*H*_3_ group (*δ* 1.2) on the side chain (d in Fig. 3). The TOCSY NMR spectrum shows also that the in-ring N*H*_2_ resonances are all strongly correlated with each other (e in Fig. 4). As well, the B*H*_2_ and B*H* groups in the ring exhibit strong correlations with the N*H*_2_ protons (f in Fig. 4) whereas the B*H* correlates more weakly with the external N*H*_2_ compared with the internal N*H*_2_ groups (g in Fig. 4). Variable temperature ^1^H{^14^N} NMR spectra of BCTB in THF-d_8_ show also the sensitivity of the N*H* chemical shifts to temperature (Fig. S9†), likely due to changes in hydrogen bonding structural motifs. Compared to the literature data for ammonia-borane and CTB,^8*d*^ we observed similar ^15^N NMR chemical shifts (Fig. S10†). However, overlapping signals at –362 ppm were assigned to the *N*H_2_-groups within the six-membered ring of BCTB (due to correlation with the N*H*_2_-protons with an integral of 6) (a in Fig. S10†) and the resonance at –367 ppm was assigned to the *exo*-cyclic *N*H_2_-group (b in Fig. S10†). Finally, the resonance at –377 ppm correlates with the *N*H_3_ resonance due to the ammonia-borane impurity in the sample (c in Fig. S10†).

### Reactivity of BCTB, **3**

**Scheme 2:**
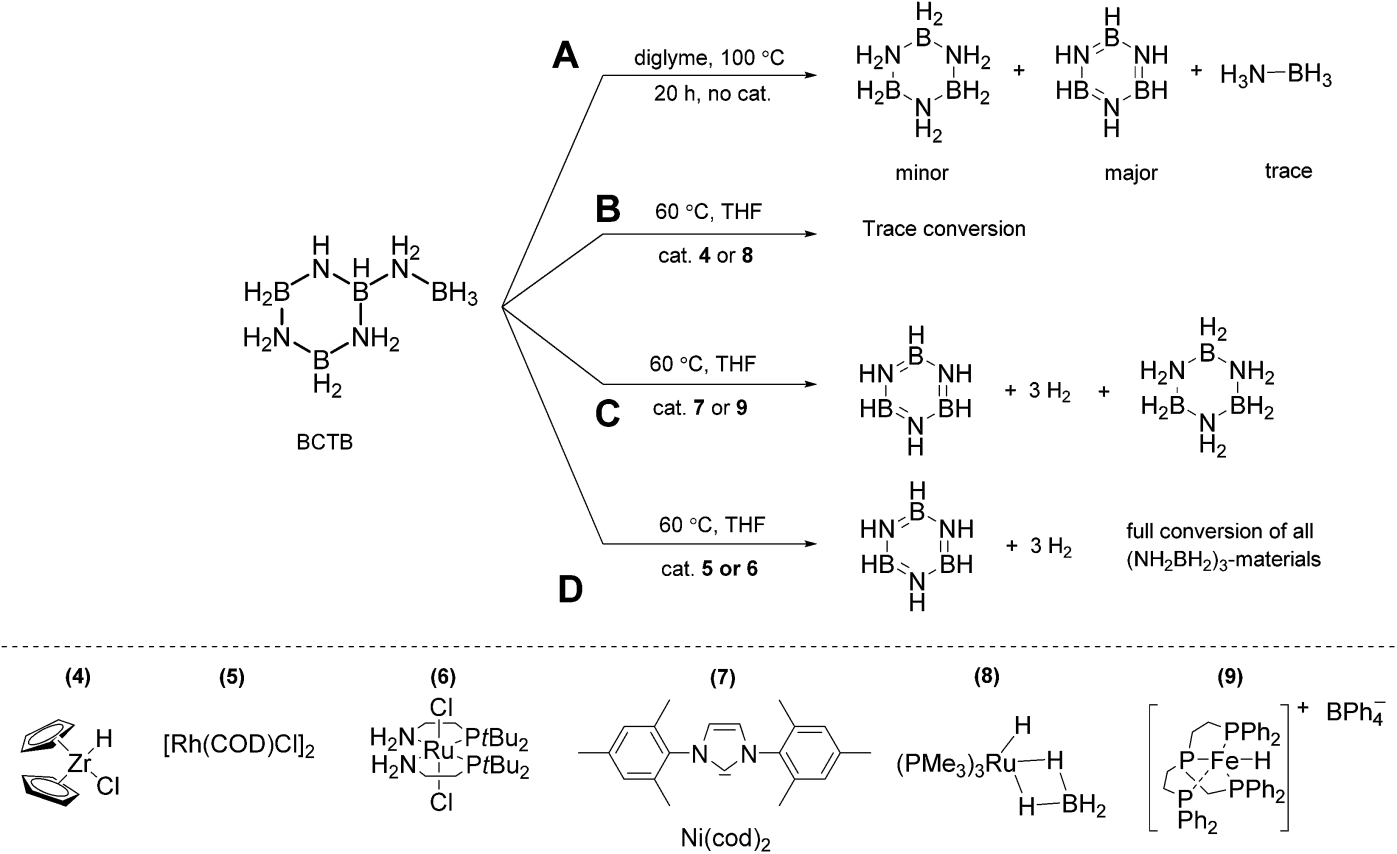
Reactivity of BCTB in the presence of complexes 4 to 9 (20 mg BCTB, 15 mol% catalyst, 2.5 mL THF, 60 °C, 16 h).

Reactivity studies demonstrate that thermolysis of **3** yields different products than those derived from its catalytic dehydrogenation. While THF or glyme solutions of AB undergo slow dehydrogenation at 85 °C, thermolysis of CTB does not proceed readily below 125 °C.^22^ Thermal treatment of BCTB (100 °C, diglyme) led to a mixture of products. After 1 and 3 h CTB, borazine and AB were detected as the main products in a sealed NMR tube experiment (Fig. S11,† Scheme 2A). After 20 h almost all BCTB was converted to borazine (as the main product) and CTB (Fig. S11†). Formation of the latter product suggests a 1,3-hydride transfer from the BH_3_ to the BH group of BCTB, affording CTB and aminoborane, NH_2_BH_2_. Indeed, when cyclohexene was used to trap the latter, a clean reaction ensued with predominate formation of CTB and NH_2_BCy_2_ (Fig. S12†). In contrast, heating BCTB at 60 °C in the presence of 7.5 mol% [Rh(μ-Cl)(cod)]_2_ (**5**, cod = 1,5-cyclooctadiene) afforded exclusively borazine and polyborazylene (Fig. S18†).

In order to probe the second dehydrogenation step of AB dehydrocoupling in more detail, we investigated a series of metal complexes as precatalysts for the dehydrogenation of BCTB. The complexes [RuCl_2_(*P*^*N*)_2_] (**6**, *P*^*N*: 2-(di-*tert*-butylphosphino)-ethylamine),^11*d*^ Ni(IMes)_2_ (**7**, IMes: *N*,*N*′-bis(2,4,6-trimethyl-phenyl)imidazol-2-ylidene),^11c,23^ and [RuH(PMe_3_)_3_(BH_4_)] (**8**)^24,25^ have been applied previously as dehydrogenation catalysts for AB. The iron hydride complex [FeH(PP_3_)][BPh_4_] (**9**, PP_3_: tris[2-(diphenylphosphino)ethyl]-phosphine)^26^ has been successfully employed for the catalytic dehydrogenation of formic acid.^4*c*^ In a typical experiment 20 mg BCTB was heated in THF (60 °C) in the presence of 15 mol% catalyst (with respect to BCTB). The reaction progress was monitored using ^11^B NMR spectroscopy with samples taken after 1, 3 and 16 h. Based on their reactivity, the catalysts can be divided into three groups (Scheme 2 **B–D**). Complexes **4** and **8** had little effect and the NMR spectra after 16 h were similar to those obtained from the control experiment (Fig. S13–15†). Complexes **7** and **9**, however, converted BCTB to a mixture of CTB, borazine and polyborazylene (Fig. S16 and S17†). The inability of complex **7** to dehydrogenate CTB to borazine was confirmed using isolated CTB (no reaction after 24 h at 60 °C). Most interestingly, a third group of catalysts, complexes **5** and **6**, effected the dehydrogenation of both BCTB and CTB after the 16 h reaction time (Fig. S18 and S19†).

## Discussion

### Synthesis and formation of BCTB, **3**

Our new synthesis of BCTB takes advantage of the sluggish reactivity of d^0^ complexes with AB^27^ and particularly the inability of Cp_2_ZrHCl to catalyse the dehydrogenation of BCTB to borazine, as shown above. We propose that the AB dehydrogenation proceeds through the amidoborane complex Cp_2_ZrCl(NH_2_BH_3_), **10**, characterized previously by Roesler *et al.* ^28^ β-elimination of the B–H bond then affords the aminoborane monomer, regenerating the catalyst. In the presence of cyclohexene, all the aminoborane was trapped by cyclohexene and no BCTB formation was observed (Fig. S20†). While we did not observe **10** in the ^11^B NMR spectra of these reactions, resonances were observed at –15.8 (quintet, *J*_B–H_ = 86 Hz) (minor) and –7.1 ppm (quintet, *J*_B–H_ = 87 Hz) due to Cp_2_Zr(BH_4_)_2_, **11**, and Cp_2_ZrCl(BH_4_), **12**, respectively (Fig. S3†). As these complexes are likely resting states of the Zr hydride catalysts, we also investigated the reaction of complex **11** with 20 equiv. of AB. Poor BCTB selectivity was observed and borazine was detected as the major product (Fig. S21†).

**Scheme 3:**
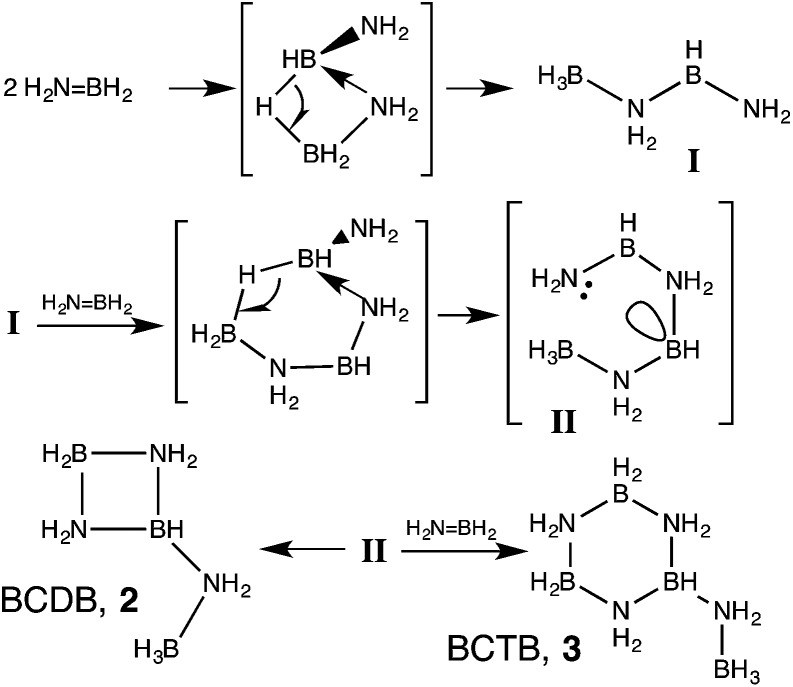
Oligomerization of aminoborane through B–H–B bridged intermediates.

In light of the variety of diverse mechanisms that have been postulated for the thermolytic and catalyzed dehydrogenation of AB, what is the origin of the extremely clean formation of BCTB with selected metal complex catalysts? Substituted primary aminoboranes such as methylaminoborane, NMeH=BH_2_, are postulated to oligomerize in solution to the cyclic trimer and, with further heating, to *N*,*N*′,*N*′′-trimethylborazine.^29^ Although the detailed mechanisms of these transformations require additional elucidation, formation of branched oligomers has been confined to reactions of the parent amine-borane, AB. In previous computational studies we^13*a*^ and others^30^ showed that this selective oligomerization could proceed *via* B–H–B bridged intermediates followed by intramolecular attack of NH_2_ on the three-coordinate boron (Scheme 3). The dimerization thus affords unusual (not yet observed) intermediate **I** with BH and BH_3_ groups. Addition of the third aminoborane monomer to **I** through a similar mechanism then generates intermediate **II** which was proposed to undergo an additional intramolecular attack of NH_2_ on three-coordinate boron to give BCDB, **2** selectively. In this work we find instead that *intermolecular* reaction of **II** with a fourth equivalent of aminoborane selectively affords the branched cyclic aminoborane *tetramer*, BCTB, **3**.

On the basis of ^11^B and ^15^N NMR spectroscopy, thermolysis of AB in glyme solvent at 80 °C was proposed to generate both cyclodiborazane and cyclotriborazane in addition to a branched aminoborane cyclic oligomer, assigned as BCDB, **2**.^8*d*^ This product distribution results presumably from the slow thermal generation of the aminoborane monomer, likely catalysed by traces of free BH_3_.^31^ In the metal-catalysed reaction, however, rapid generation of aminoborane favors selective generation of BCTB, **3**, as long as the monomer does not take part in the metal coordination oligomerization pathway to linear polyamino-borane.^16^ Further work is thus needed to fully characterize the branched aminoborane cyclic oligomer derived from uncatalysed AB themolysis.

### Thermal *vs.* catalysed conversion of BCTB, **3**

The branched aminoborane tetramer, BCTB, **3**, is considerably more reactive than the cyclic trimer, CTB, **1**, which is believed to undergo competing ring-opening and dehydrogenation pathways.^22*b*^ The formation of some borazine and ammonia-borane from the thermolysis of **3** can be attributed to hydrogen transfer processes (eqn (1)) as studied recently by Manners and co-workers.^32^ Consistent with this proposal, neither AB nor borazine was observed on thermolysis of **3** in the presence of cyclohexene which effectively traps the aminoborane monomer.^33^

1

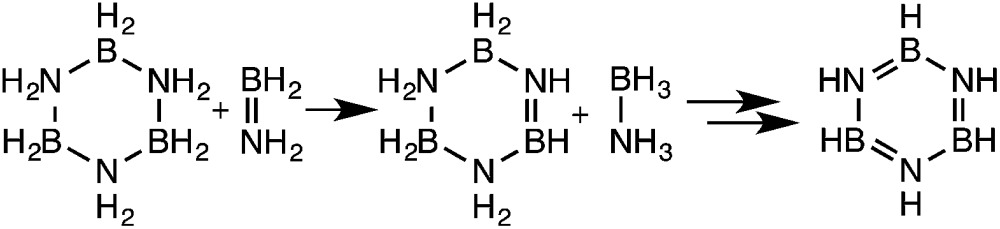




The catalysed reactions of **3** shed additional light on the second H_2_ release step from AB. The fact that Ru catalyst precursor **8** is unable to dehydrogenate **3** is consistent with previous studies that showed the sensitivity of **8** to the reaction solvent. While poor selectivity for AB dehydrogenation was observed in THF, formation of insoluble polyaminoborane could be prevented using ionic liquids with nucleophilic anions.^25^ Similar observations were made using the Ni(NHC)_2_ catalyst that required a mixed benzene–glyme solvent system to release >2 equiv. of H_2_.^11*c*^ In spite of the short lifetime of this catalyst, the small amount of CTB remaining in Fig. 1(d) supports the greater reactivity seen for **3** *vs.* CTB using the similar catalyst **7**. In contrast, the [RuCl_2_(*P*^*N*)_2_]/KOBu^*t*^ catalyst system **6** that was reported to afford a single equivalent of hydrogen from AB readily converted both CTB and **3** to borazine and polyborazylene. This would thus be a good candidate for further solvent studies to avoid the aminoborane coordination polymerization pathway from AB. Finally, the Rh colloids^12a,b^ or clusters^34^ derived from the chlororhodium diene catalyst precursor **5** are also effective catalysts for conversion of **3** to borazine and polyborazylene although catalyst lifetime experiments have yet to be performed.^12*b*^

## Conclusions

Utilization of the Cp_2_ZrHCl catalyst precursor has allowed for the isolation and characterization of the key intermediate in the second step of metal-catalysed AB dehydrogenation. In contrast to previous assignments, the intermediate has now been identified as the BN ethylcyclohexane analog, the branched, cyclic aminoborane *tetramer*, B-(cyclotriborazanyl)amine-borane (**3**). Reactivity studies show that higher yields of **3** would likely be obtained if reaction of the aminoborane monomer with CTB to give borazine and AB (eqn (1)) could be controlled. Testing of several catalysts commonly used for dehydrocoupling reactions for this second dehydrogenation step revealed an important difference in selectivity as some catalysts converted **3**, *but not CTB*, into borazine and polyborazylene. With this information in hand we are now poised to address the challenging third step of AB dehydrogenation – identifying an effective catalyst for the BN cross-linking of borazine to polyborazylene.^35^
