# Supplementary material for: Probing the second dehydrogenation step in ammonia-borane dehydrocoupling: characterization and reactivity of the key intermediate, B-(cyclotriborazanyl)amine-borane
Source: Chem Sci. 2014 Oct 30;6(1):618–24. doi: 10.1039/c4sc02710h (PMC5491959; doi:10.1039/c4sc02710h)
Supplement: Supplementary file 1 [file SC-006-C4SC02710H-s001.pdf]

Electronic Supplementary Material (ESI) for Chemical Science.
This journal is © The Royal Society of Chemistry 2014

Supplementary Information

Probing the second dehydrogenation step in ammonia-borane dehydrocoupling: Characterization and reactivity of the key intermediate, B-(cyclotriborazanyl)amine-borane

Hassan A. Kalviri[a], Felix Gärtner,[a,b] Gang Ye[a], Ilia Korobkov[a] and R. Tom Baker[a]*

[a] Department of Chemistry and Centre for Catalysis Research and Innovation, University of Ottawa, 30 Marie Curie, Ottawa, ON, K1N 6N5 CANADA.

[b] Leibniz-Institut für Katalyse, Albert-Einstein Straβe 29a, 18059 Rostock, GERMANY.

Contents:

- General information and instrumentation…..……………………………….............. 2
- X-ray crystallography……………………………………………………………….. 2
- Preparation of BCTB from AB + 11 mol% ([$FeH(CH_2PMe_2)(PMe_3)_3$]……………….. 3
- $^{31}P\{^1H\}$ NMR spectrum of [$FeH(CH_2PMe_2)(PMe_3)_3$] (Fig. S1)……………………… 4
- $^{11}B\{^1H\}$ NMR spectra of sublimed BCTB from AB reaction with [$FeH(CH_2PMe_2)$-($PMe_3)_3$] (Fig. S2)…………………………………………………………………….... 4
- Preparation of BCTB from AB + 5 wt% [Cp_2ZrHCl]………………………………… 5
- $^{11}B\{^1H\}$ NMR spectra of BCTB in the presence of [$(Cp)_2ZrHCl$] (Fig. S3)………….. 6
- ^{11}B (top) and $^{11}B\{^1H\}$ (bottom) NMR spectra (THF/C_6D_6) of BCTB with assignments (Fig. S4)…………………………………………………………………………….... 6
- $^{11}B\{^1H\}$ NMR spectrum (THF/C_6D_6) of BCTB with integrals (Fig. S5)……………... 7
- $^1H\{^{11}B\}$ NMR spectrum (THF-d_8) of BCTB with integrals (Fig. S6)…..…………….. 7
- ORTEP view of BCTB (**3**) co-crystallized with 18-crown-6 (Fig. S7)……..…...….… 8
- Crystal data and structure refinement (Table S1)…………………………………… 9
- Bond lengths [Å] and angles [°] for **3**•18-crown-6 (Table S2)……………………… 10
- Torsion angles [°] for **3**•18-crown-6 (Table S3)………………………………….. 11
- Bond lengths [Å] and angles [°] for **3**•glyme (Table S4)... 12
- Torsion angles [°] for **3**•glyme (Table S5)…………….. 13
- Hydrogen and dihydrogen bonds for BCTB **3**•glyme [Å and °] (Table S6)……………… 13
- ORTEP unit cell view of **3**•glyme (Fig. S8)….………………………………….... 14
- Variable temperature $^1H\{^{14}N\}$ NMR spectra (THF-d_8) of BCTB (Fig. S9)………….... 15
- 2D-NMR (^1H-^{15}N-HSQC) (THF-d_8) of BCTB (Fig. S10)….. 15
- Thermal decomposition of BCTB in diglyme……………………………………….. 16
- Thermal reaction of BCTB in diglyme at 100 °C (Fig. S11)…..................................... 16
- $^{11}B\{^1H\}$ NMR spectra of BCTB in the presence of cyclohexene (Fig. S12)................. 17
- Catalytic decomposition of BCTB…………………………………………………... 17
- $^{11}B\{^1H\}$ NMR spectra of BCTB thermolysis (60 °C) (Fig. S13)…………….……….. 18
- $^{11}B\{^1H\}$ NMR spectra of BCTB + 15 mol% [$(Cp)_2ZrHCl$] (Fig. S14)………………... 18
- $^{11}B\{^1H\}$ NMR spectra of BCTB + 15 mol% [$RuH(PMe_3)_3(BH_4)$] (Fig. S15)………… 19
- $^{11}B\{^1H\}$ NMR spectra of BCTB + 15 mol% [$Ni(COD)_2$] /2 NHC- (Fig. S16)………... 19
- $^{11}B\{^1H\}$ NMR spectra of BCTB + 15 mol% [$Fe(PP_3)H][BPh_4$] (Fig. S17)……….…. 20

- $^{11}B\{^1H\}$ NMR spectra of BCTB + 7.5 mol% $[Rh(COD)Cl]_2$ (Fig. S18)…………………....... 20
- $^{11}B\{^1H\}$ NMR spectra of BCTB + 15 wt% $[Ru(N^\wedge P)_2Cl_2]$ / KO(*t*-Bu) (Fig. S19)…......... 21
- ^{11}B NMR spectra of the reaction mixture from AB + 5 mol% $[Cp_2ZrHCl]$ in the presence of cyclohexene (Fig. S20)…………………………... 21
- $^{11}B\{^1H\}$ NMR spectra of the reaction mixture from AB + 5 mol% $[Cp_2ZrHCl]$ (top) and $Cp_2Zr(BH_4)_2$ (bottom) at 50 °C after 48 h. (Fig. S21).. 22
- References……………………………………………………………………....... 22

General information and instrumentation: Experiments were conducted under nitrogen, using Schlenk techniques or an MBraun glove box. Ammonia-borane and Cp_2ZrHCl were purchased from GFS Chemicals and Aldrich, respectively and were used under air/moisture-free conditions without further purification. Tetrahydrofuran (THF) and glyme were dried on columns of activated alumina using a J. C. Meyer (formerly Glass Contour®) solvent purification system. Benzene-d$_6$ (C_6D_6) was dried by standing over activated alumina overnight, followed by filtration. A Bruker AVANCE 500 MHz spectrometer equipped with a TBO probe and a Bruker AVANCE II 300 MHz spectrometer equipped with a BBO probe were used for the NMR experiments. ^1H NMR spectra were referenced to the residual proton peaks associated with the deuterated solvents (C_6D_6: 7.16 ppm). $^{11}B\{^1H\}$ NMR data were referenced to external $BF_3.Et_2O$ set to 0.0 ppm. ^{15}N NMR spectra were referenced to $MeNO_2$ at 0.0 ppm

X-ray crystallography: Data collection results for compounds **tb055** and **tb061** represent the best data sets obtained in several trials for each sample. The crystals were mounted on thin glass fibers using paraffin oil. Prior to data collection crystals were cooled to 200 K. Data were collected on a Bruker AXS KAPPA single-crystal diffractometer equipped with a sealed Mo tube source (wavelength 0.71073 Å) APEX II CCD detector. Raw data collection and processing were performed with APEX II software package from BRUKER AXS.[1] Diffraction data for **tb055** sample was collected with a sequence of 0.3° ω scans at 0, 120, and 240° in φ. Due to lower unit cell symmetry in order to ensure adequate data redundancy, diffraction data for **tb061** was collected with a sequence of 0.3° ω scans at 0, 90, 180 and 270° in φ. Initial unit cell parameters were determined from 60 data frames with 0.3° ω scan each collected at the different sections of the Ewald sphere. Semi-empirical absorption corrections based on equivalent reflections were applied.[2] Systematic absences in the diffraction data-set and unit-cell parameters were consistent with triclinic $\mathbf{P}\bar{1}$ (№2) for compounds **tb061** and monoclinic $\mathbf{P}2_1/n$ (№14, alternative settings) for compounds **tb055**. Solutions in the centrosymmetric space groups for all compound yielded chemically reasonable and computationally stable results of refinement. The structures were solved by direct methods, completed with difference Fourier synthesis, and refined with full-matrix least-squares procedures based on F^2.

The structure of **tb055** contained one target compound molecule situated in the general position of the unit cell and two more molecules of 18-crown-6 located in two different inversion centers. The structural model for **tb061** contains only one molecule of target compound located in the general position.

For both compounds all hydrogen atoms positions were calculated based on the geometry of the related non-hydrogen atoms. All hydrogen atoms were treated as idealized contributions during the refinement. All scattering factors are contained in several versions of the SHELXTL program library, with the latest version used being v.6.12.[3] Additional crystallographic data and selected data collection parameters are detailed in Tables S1-5 and the cif files for the structures of **3·glyme** and **3·18-crown-6** have been deposited.

Preparation of BCTB from AB + 11mol % ([FeH(CH$_2$PMe$_2$)(PMe$_3$)$_3$][4]

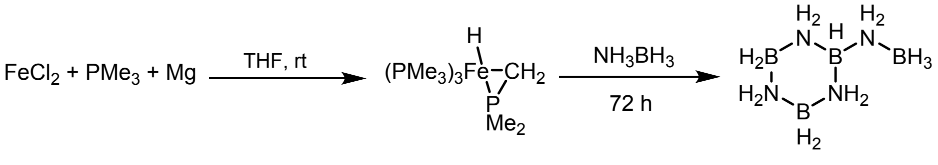

In a nitrogen-filled glove box PMe$_3$ (500 mg, 6.6 mmol, 4.5 equiv.) in THF (5 mL) was added to a vigorously stirred mixture of FeCl$_2$ (186 mg, 1.46 mmol, 1.0 equiv.) in THF (10 mL). Initially, the reaction mixture turned deep red, and the color faded to green within several minutes. Then Mg (35.5 mg, 1.46 mmol, 1.0 equiv.) was added and the resulting mixture was stirred until all the Mg was consumed. Depending on the reactivity of the Mg, the reaction time may take between 6-24 h. The start of the reduction can be observed by a color change to dark yellow. Prolonged reaction times led to formation of a black precipitate. When all Mg was consumed, the dark yellow mixture was filtered through Celite and the $^{31}P\{^1H\}$ NMR spectrum (Figure S1) indicated formation of [FeH(CH$_2$PMe$_2$)(PMe$_3$)$_3$] (major) and FeH$_2$(PMe$_3$)$_4$ (minor). The filtered solution was added gradually to a solution of AB (400 mg, 13.0 mmol) in THF (*ca.* 50 mL). The resulting bright yellow solution was stirred at room temperature for 72 h. Within 30 min a white precipitate was formed and the color of the suspension turned pale yellow. After 72 h the suspension was filtered to afford 240 mg of colorless, THF-insoluble polyaminoborane[2] (yield from AB = 64%) and the volume of the yellow solution was reduced to *ca.* 10 mL. The soluble BCTB product was then precipitated out by drop-wise addition of toluene to the concentrated solution. The colorless solid was washed with toluene and diethyl ether and dried to yield BCTB (53 mg, 19 %). This crude material was purified further by vacuum sublimation at 80 °C and 2 x10^{-6} torr for 5 d to remove any CTB and unreacted AB (Figure S2).

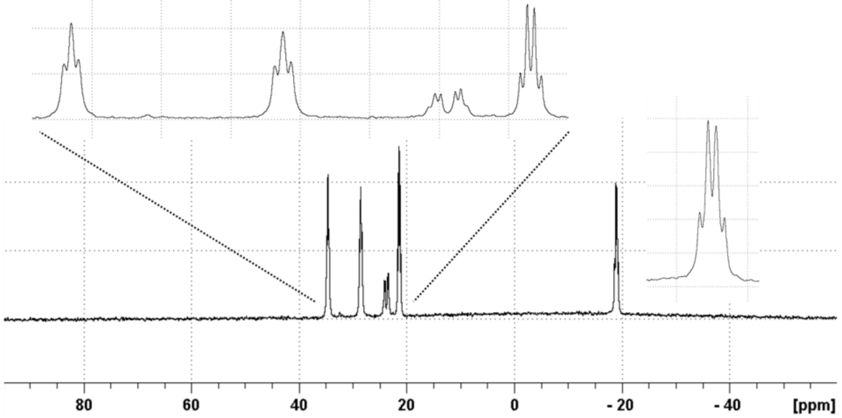

Figure S1. $^{31}P\{^1H\}$ NMR spectrum of $[FeH(CH_2PMe_2)(PMe_3)_3]$ (in a mixture with the dihydride $[FeH_2(PMe_3)_4]$) formed *in situ* from $FeCl_2$, PMe_3 and Mg in THF.[1]

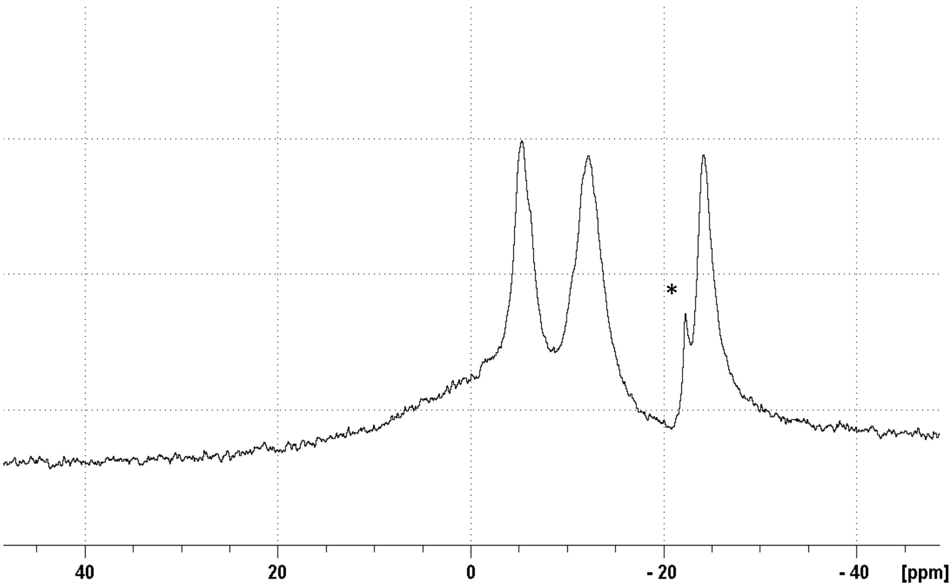

Figure S2. $^{11}B\{^1H\}$ NMR spectrum of sublimed BCTB, with minor AB impurity(*), obtained from the reaction of AB + 11 mol% $[FeH(PMe_2CH_2)(PMe_3)_3]$.

Preparation of BCTB from AB + 5 mol% [Cp_2ZrHCl]

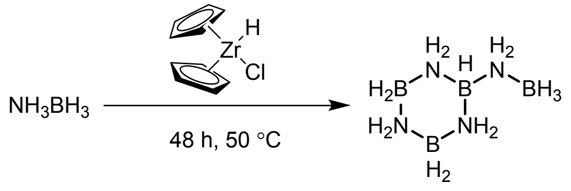

In a nitrogen-filled glove box AB (1.10 g, 35.6 mmol) was added to [Cp_2ZrHCl] (466 mg, 1.8 mmol, 5 mol%) in THF (40 mL). The colorless mixture was stirred at 50 °C for 48 h. During the course of the reaction a small amount of colorless precipitate formed, which was removed by filtration. All volatiles were then removed and the remaining colorless solid was washed with toluene several times. The product was purified further (in order to remove AB and CTB) by sublimation at 80 °C and $2x10^{-6}$ torr for 24 h (3×8 h), yielding 410 mg of BCTB as a colorless solid (53 %) (Figure S3). $^{1}H\{^{11}B\}$ NMR (THF-*d8,* -15 °C): δ 3.2, 3.0 (3H, overlapping ring NH_2); 2.7 (2H, exo NH_2); 2.5 (1H, BH); 2.2 and 2.1 (4H, axial and equatorial BH_2 hydrogens); 1.2 (3H, BH_3). ^{11}B NMR (C_6D_6): -5.5 (d, BH, J_{BH}: 114 Hz); -11.4 (t, BH_2, J_{BH}: 96 Hz); -24.5 ppm (q, BH_3, J_{BH} : 90 Hz). ^{15}N NMR (indirect measurement, THF-*d8*): -362 (3N, overlapping ring NH_2); -367 ppm (1N, exo NH_2).

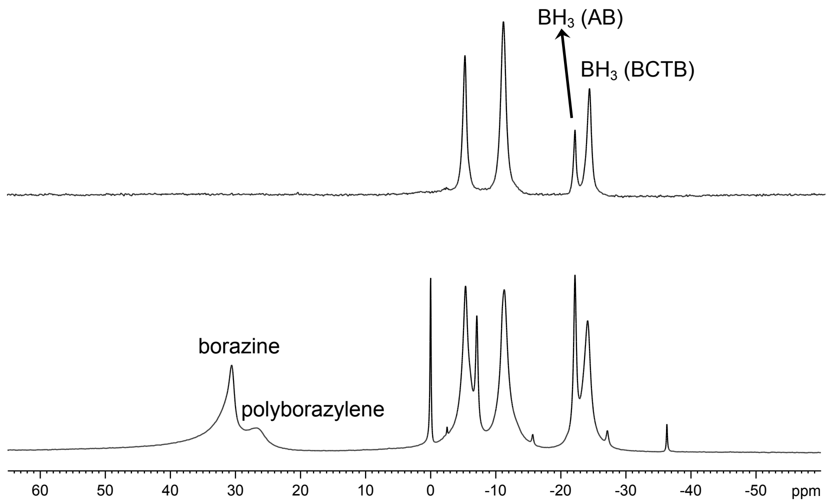

Figure S3. $^{11}B\{^1H\}$ NMR spectra (THF/C_6D_6) of the reaction mixture from AB + 5 mol% [Cp_2ZrHCl] before (bottom; resonance at 0.0 ppm is due to $BF_3{\cdot}OEt_2$ standard in capillary) and after purification (top; resonance at -22.2 ppm is due to AB).

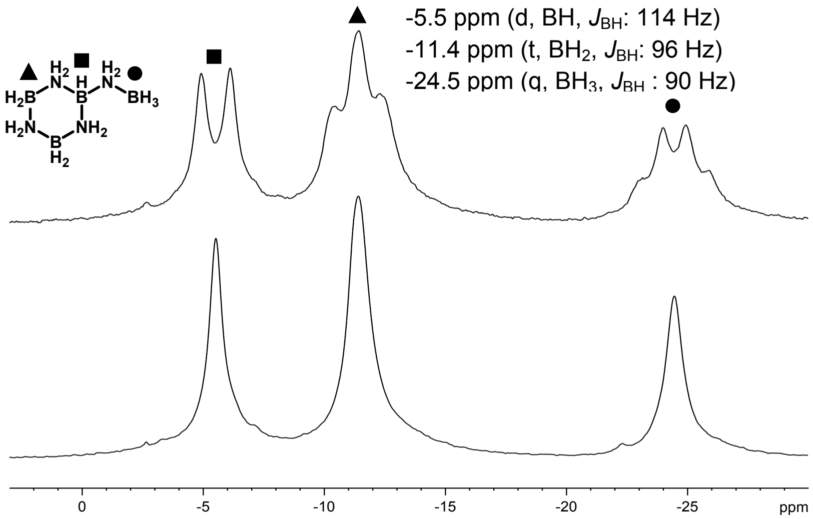

Figure S4. ^{11}B (top) and $^{11}B\{^1H\}$ (bottom) NMR spectra (THF/C_6D_6) of purified BCTB with corresponding assignments.

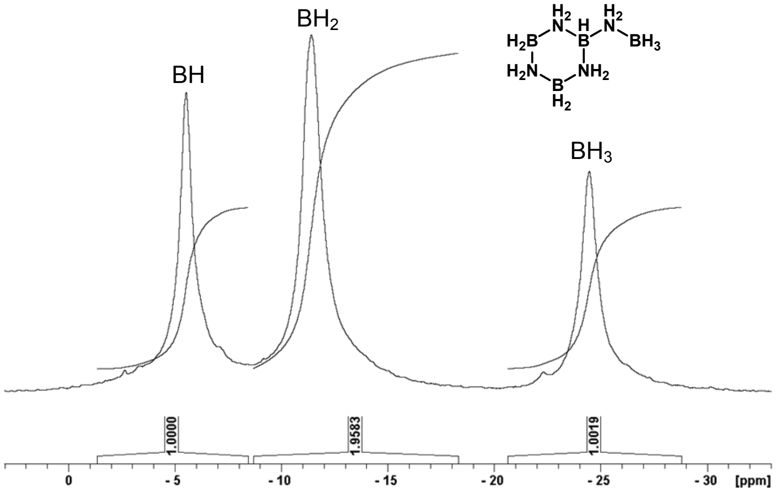

Figure S5. $^{11}B\{^{1}H\}$ NMR spectrum (THF/C_6D_6) of purified BCTB with integrals.

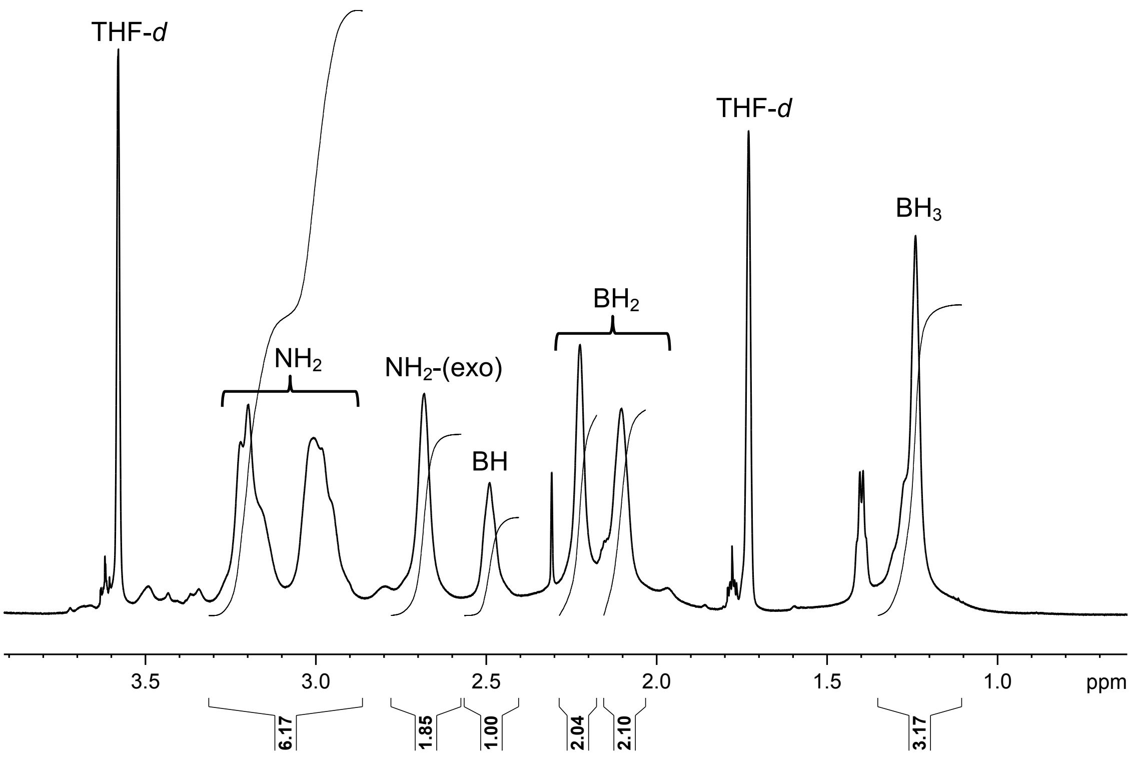

Figure S6. $^{1}H\{^{11}B\}$ NMR spectrum (THF-d_8, -15 °C) of purified BCTB with integrals. Some impurities are visible underneath the BH_2 peak at 2.1 ppm (related to CTB) and BH_3 peak at 1.2 ppm.

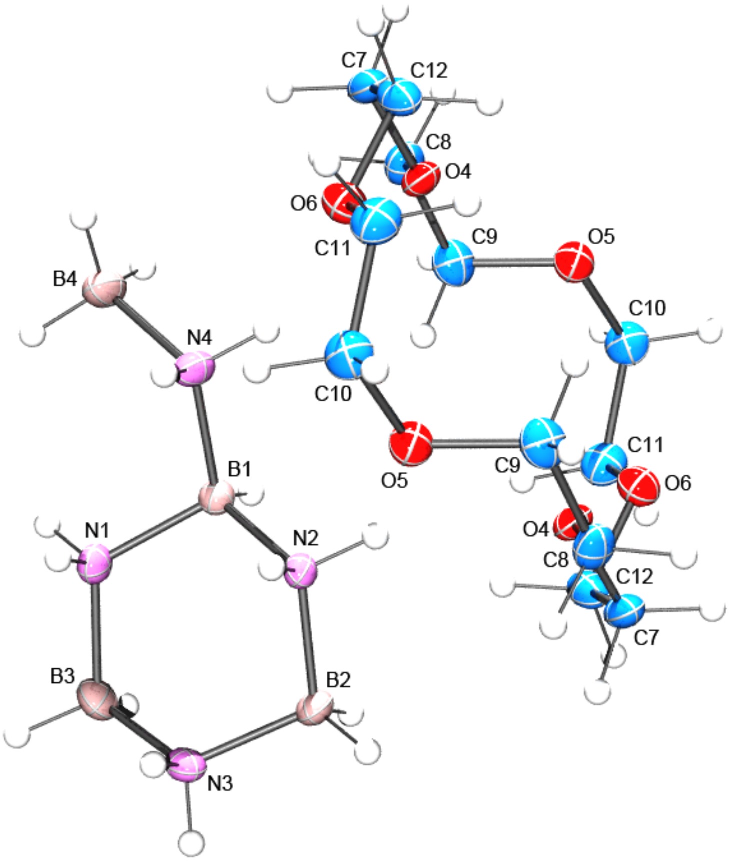

Figure S7. ORTEP view of BCTB (**3**) co-crystallized with 18-crown-6. The thermal ellipsoids are drawn at the 50% probability level.

Table S1. Crystal data and structure refinement

Complex	**3**•18-crown-6	**3**•glyme
ID code	tb055	tb061
Formula	$C_{12}H_{40}B_4N_4O_6$	$C_5H_{26}B_3N_4O_2$
Mw	379.72	206.73
Color	colorless	colorless
Temp (K)	200(2)	200(2)
Crystal system	Monoclinic	Triclinic
Space group	P 2(1)/n	P-1
a/Å	16.026(3)	6.9419(7)
b/ Å	8.7017(15)	8.8077(8)
c/ Å	17.485(3)	12.4771(12)
α/^{o}	90.00	91.790(3)
β/^{o}	115.469(6)	103.761(3)
γ/^{o}	90.00	11.731(3)
V/Å^3	2201.5(7)	680.63(11)
Z	4	2
Dc/g cm^{-1}	1.146	1.009
μ/mm^{-1}	0.084	0.070
F(000)	832	230
Crystal size/mm	0.15×0.13×0.12	0.21×0.16×0.15
2θ range/^{o}	1.44-24.11	1.70-28.34
	-18<h<18	-9<h<9
Index range	-10<k<10	-11<k<11
	-20<l<20	-16<l<16
Indep. reflns collected/unique	26868/3449	10580/3314
Max. and min. transmission	0.9900 and 0.9875	0.9896 and 0.9855
(Rint)	0.0646	0.0447
R1, wR2 (I>2θ(I))	0.0568, 0.1214	0.0619, 0.1563
R1, wR2(all data)	0.0752, 0.1302	0.0844, 0.1711
Goodness of fit, F^2	1.063	1.049
Data/restraints/params	3449/0/235	3314/0/127
Largest diff. peak, hole/Å^3	0.255, -0.173	0.340, -0.206

Table S2. Bond lengths [Å] and angles [°] for **3**•18-crown-6

B(1)-N(1)	1.562(3)
B(1)-N(2)	1.550(3)
B(1)-N(4)	1.562(3)
B(2)-N(3)	1.573(3)
B(2)-N(2)	1.591(3)
B(3)-N(3)	1.567(4)
B(3)-N(1)	1.591(3)
B(4)-N(4)	1.595(3)
O(1)-C(2)	1.430(3)
O(1)-C(1)	1.434(3)
O(2)-C(3)	1.421(3)
O(2)-C(4)	1.431(3)
O(3)-C(5)	1.415(3)
O(3)-C(6)	1.417(3)
O(4)-C(7)	1.414(3)
O(4)-C(8)	1.431(3)
O(5)-C(9)	1.416(3)
O(5)-C(10)	1.420(3)
O(6)-C(12)	1.416(3)
O(6)-C(11)	1.414(3)
C(1)-C(6)#1	1.498(4)
C(2)-C(3)	1.484(4)
C(4)-C(5)	1.506(4)
C(6)-C(1)#1	1.498(4)
C(7)-C(12)#2	1.488(4)
C(8)-C(9)	1.490(4)
C(10)-C(11)	1.497(4)
C(12)-C(7)#2	1.488(4)
N(1)-B(1)-N(2)	106.58(18)
N(1)-B(1)-N(4)	107.80(18)
N(2)-B(1)-N(4)	111.96(19)
N(3)-B(2)-N(2)	106.99(18)
N(3)-B(3)-N(1)	106.5(2)
B(1)-N(1)-B(3)	117.17(19)
B(1)-N(2)-B(2)	115.60(18)
B(2)-N(3)-B(3)	114.03(19)
B(1)-N(4)-B(4)	116.98(19)
C(2)-O(1)-C(1)	113.95(19)
C(3)-O(2)-C(4)	113.62(19)
C(5)-O(3)-C(6)	113.0(2)
C(7)-O(4)-C(8)	111.68(18)
C(9)-O(5)-C(10)	114.63(19)
C(12)-O(6)-C(11)	112.33(19)
O(1)-C(1)-C(6)#1	113.9(2)
O(1)-C(2)-C(3)	110.3(2)
O(2)-C(3)-C(2)	110.1(2)
O(2)-C(4)-C(5)	113.3(2)

O(3)-C(5)-C(4)	108.6(2)
O(3)-C(6)-C(1)#1	110.7(2)
O(4)-C(7)-C(12)#2	109.8(2)
O(4)-C(8)-C(9)	109.7(2)
O(5)-C(9)-C(8)	110.1(2)
O(5)-C(10)-C(11)	114.6(2)
O(6)-C(11)-C(10)	109.4(2)
O(6)-C(12)-C(7)#2	110.7(2)

Symmetry transformations used to generate equivalent atoms:
#1 -x,-y+1,-z   #2 -x+1,-y+1,-z

Table S3. Torsion angles [°] for **3**•18-crown-6

N(2)-B(1)-N(1)-B(3)	-54.0(3)
N(4)-B(1)-N(1)-B(3)	-174.4(2)
N(3)-B(3)-N(1)-B(1)	55.1(3)
N(1)-B(1)-N(2)-B(2)	54.5(2)
N(4)-B(1)-N(2)-B(2)	172.19(19)
N(3)-B(2)-N(2)-B(1)	-57.3(3)
N(2)-B(2)-N(3)-B(3)	57.7(3)
N(1)-B(3)-N(3)-B(2)	-56.0(3)
N(1)-B(1)-N(4)-B(4)	-59.6(3)
N(2)-B(1)-N(4)-B(4)	-176.5(2)
C(2)-O(1)-C(1)-C(6)#1	82.5(3)
C(1)-O(1)-C(2)-C(3)	161.6(2)
C(4)-O(2)-C(3)-C(2)	162.2(2)
O(1)-C(2)-C(3)-O(2)	68.3(3)
C(3)-O(2)-C(4)-C(5)	-74.2(3)
C(6)-O(3)-C(5)-C(4)	173.7(2)
O(2)-C(4)-C(5)-O(3)	-58.0(3)
C(5)-O(3)-C(6)-C(1)#1	-171.6(2)
C(8)-O(4)-C(7)-C(12)#2	173.2(2)
C(7)-O(4)-C(8)-C(9)	176.4(2)
C(10)-O(5)-C(9)-C(8)	173.1(2)
O(4)-C(8)-C(9)-O(5)	69.5(3)
C(9)-O(5)-C(10)-C(11)	64.6(3)
C(12)-O(6)-C(11)-C(10)	-162.3(2)
O(5)-C(10)-C(11)-O(6)	56.9(3)
C(11)-O(6)-C(12)-C(7)#2	178.1(2)

Symmetry transformations used to generate equivalent atoms:
#1 -x,-y+1,-z   #2 -x+1,-y+1,-z

Table S4. Bond lengths [Å] and angles [°] for **3**•glyme

N(1)-B(1)	1.556(2)
N(1)-B(3)	1.580(2)
N(2)-B(1)	1.560(2)
N(2)-B(2)	1.588(2)
N(3)-B(3)	1.572(2)
N(3)-B(2)	1.572(2)
N(4)-B(1)	1.5736(19)
N(4)-B(4)	1.5883(19)
O(1)-C(2)	1.410(3)
O(1)-C(1)	1.414(2)
O(2)-C(3)	1.413(2)
O(2)-C(4)	1.423(2)
C(2)-C(3)	1.495(3)
B(1)-N(1)-B(3)	115.64(12)
B(1)-N(2)-B(2)	114.74(13)
B(3)-N(3)-B(2)	116.83(12)
B(1)-N(4)-B(4)	118.81(10)
C(2)-O(1)-C(1)	112.08(18)
C(3)-O(2)-C(4)	112.45(15)
N(1)-B(1)-N(2)	106.71(11)
N(1)-B(1)-N(4)	109.48(12)
N(2)-B(1)-N(4)	108.92(11)
N(3)-B(2)-N(2)	106.76(13)
N(3)-B(3)-N(1)	107.10(11)
O(1)-C(2)-C(3)	109.38(18)
O(2)-C(3)-C(2)	109.14(17)

Table S5. Torsion angles [°] for **3•glyme**

B(3)-N(1)-B(1)-N(2)	-57.82(14)
B(3)-N(1)-B(1)-N(4)	-175.54(10)
B(2)-N(2)-B(1)-N(1)	58.43(15)
B(2)-N(2)-B(1)-N(4)	176.52(11)
B(4)-N(4)-B(1)-N(1)	56.43(16)
B(4)-N(4)-B(1)-N(2)	-59.90(16)
B(3)-N(3)-B(2)-N(2)	53.00(18)
B(1)-N(2)-B(2)-N(3)	-55.89(17)
B(2)-N(3)-B(3)-N(1)	-52.16(17)
B(1)-N(1)-B(3)-N(3)	54.36(15)
C(1)-O(1)-C(2)-C(3)	-179.12(18)
C(4)-O(2)-C(3)-C(2)	178.9(2)
O(1)-C(2)-C(3)-O(2)	-67.7(3)

Table S6. Hydrogen and dihydrogen bonds for **3•glyme** [Å and °]

D-H...A	d(D-H)	d(H...A)	d(D... A)	<(DHA)
N(4)-H(4B)...O(2)	0.99	2.06	2.9891(18)	155.3
N(4)-H(4A)...H(4C)#1	0.99	2.27	3.181(2)	153.0
N(1)-H(1A)...H(3C)#2	0.99	2.12	2.998(2)	146.7
N(1)-H(1B)...H(4E)#3	0.99	2.26	3.117(2)	144.5
N(3)-H(3A)...H(4D)#3	0.99	2.08	3.067(2)	173.3

Symmetry transformations used to generate equivalent atoms:
#1 -x+1,-y,-z+2 #2 -x+1,-y+1,-z+2 #3 -x,-y,-z+2

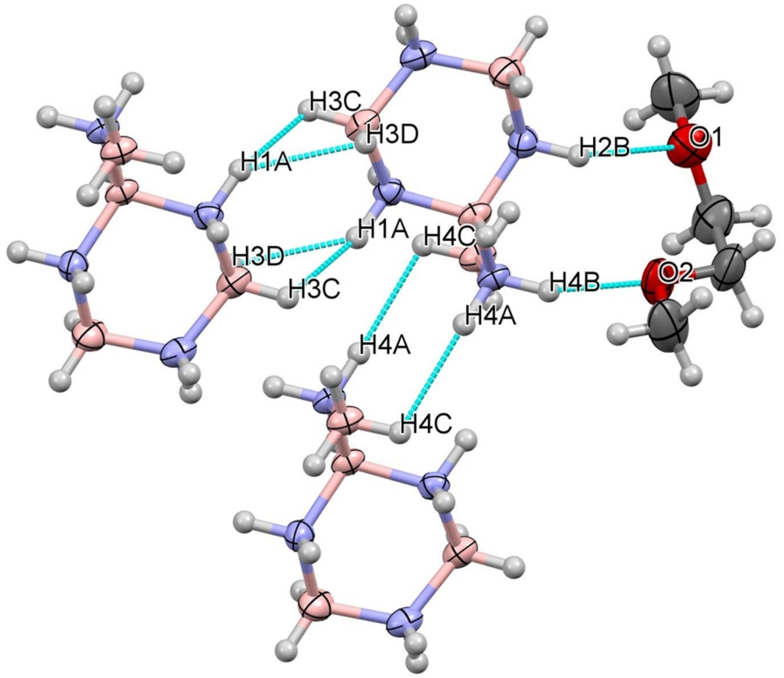

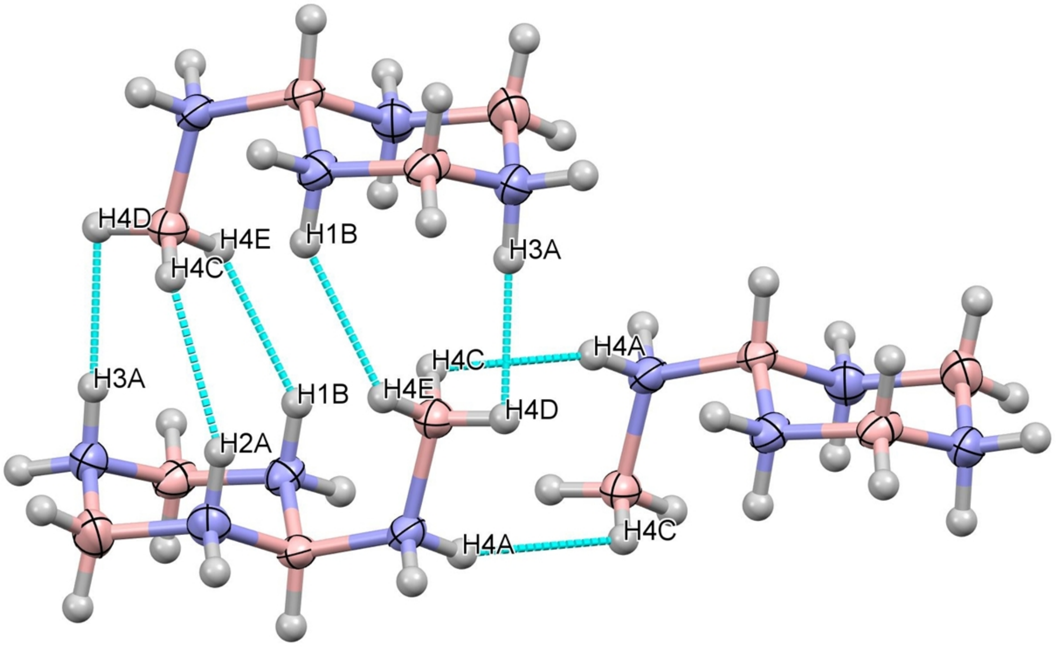

Figure S8. Two views of hydrogen- and dihydrogen bonding in **3**•glyme

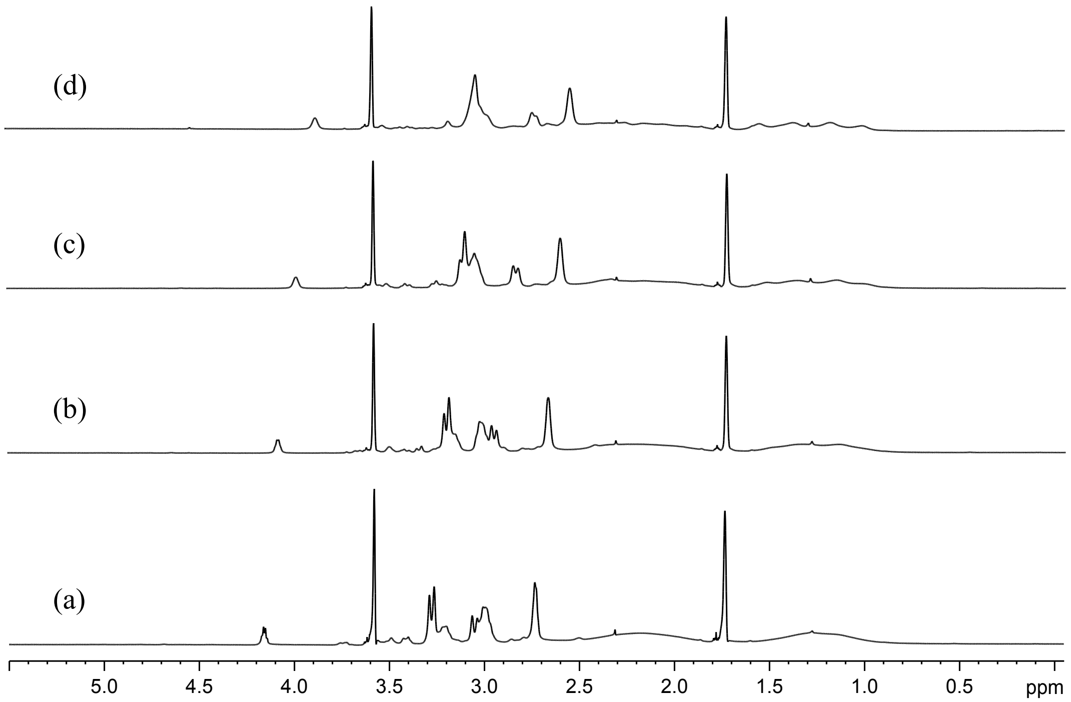

Figure S9. Variable temperature $^{1}H\{^{14}N\}$ NMR spectra (THF-d_8) of BCTB at -35 °C (a), -10 °C (b), 20 °C (c) and 55 °C (d) (due to ^{11}B quadrupolar coupling, BH peaks are observed as broad signals at 1.25 and 2.20 ppm).

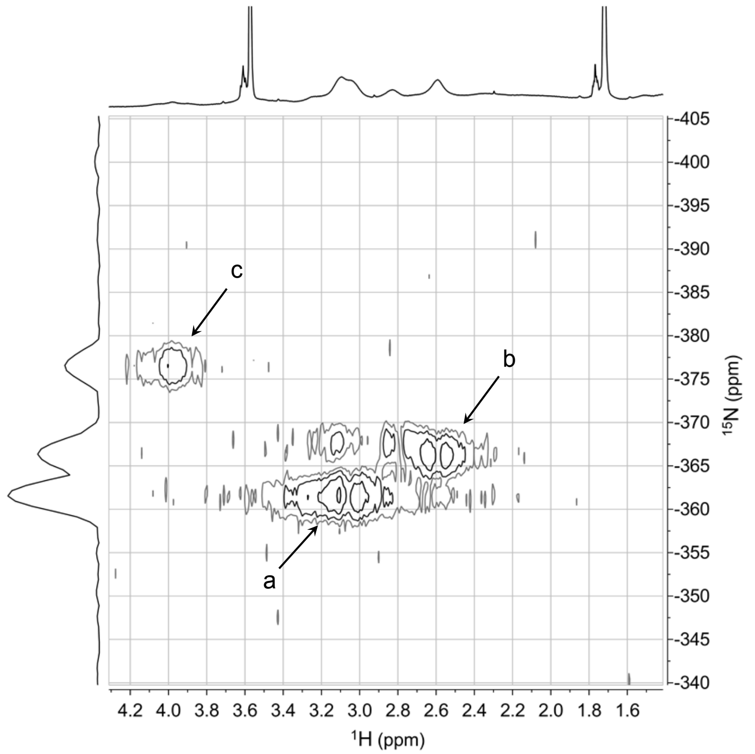

Figure S10. 2D-NMR (^{1}H-^{15}N-HSQC) (THF-d_8) of BCTB containing AB impurity.

Thermal decomposition of BCTB in diglyme

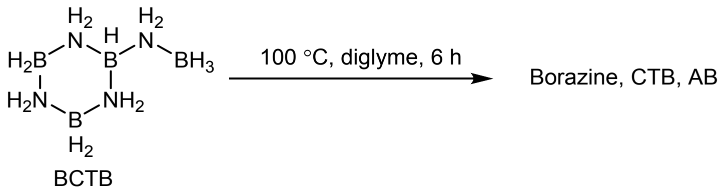

In a nitrogen-filled glove box BCTB (12 mg) was dissolved in diglyme (0.5 mL) and 0.1 mL C_6D_6. The resulting solution was transferred to a J. Young NMR tube and the head-space was removed *in vacuo* while the contents of the NMR tube were frozen with liquid nitrogen. The tube was then sealed and transferred to a preheated bath (100 °C). The reaction mixture was cooled to room temperature after 1.5, 3, 6, and 20 h for NMR analysis, after which the NMR tube was returned to the heating bath.

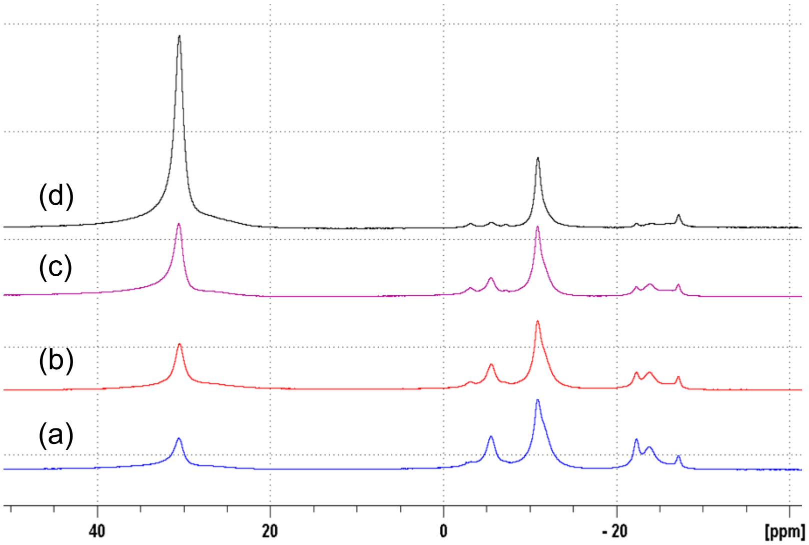

Figure S11. Thermal reaction of BCTB in diglyme at 100 °C. $^{11}B\{^1H\}$ NMR spectra (THF/C_6D_6) at t = 1.5 (a), 3.0 (b), 6.0 (c) and 20 h (d).

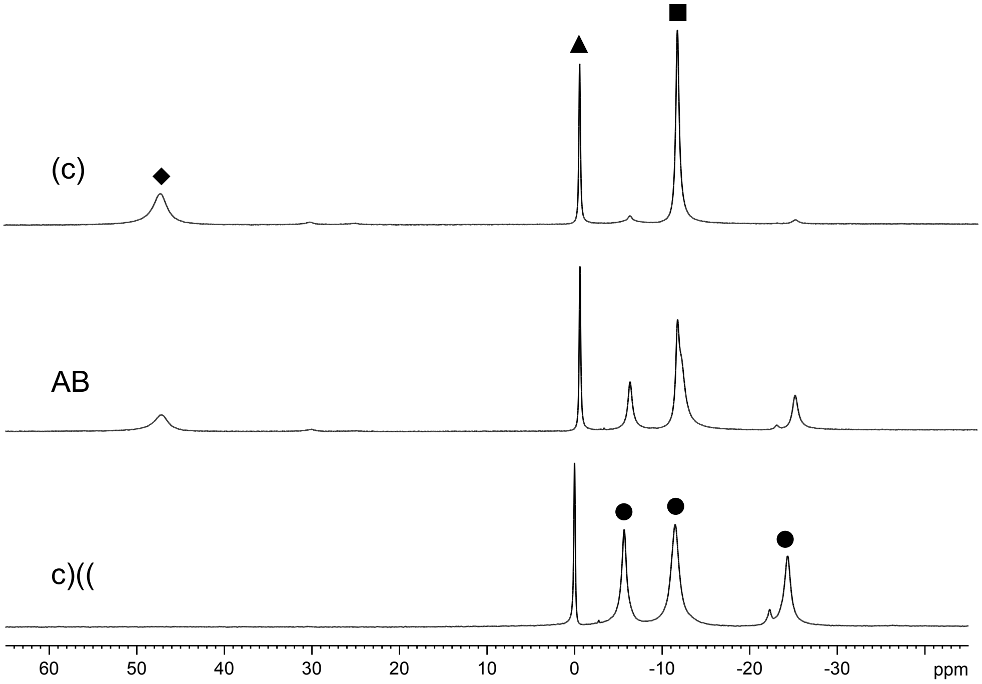

Figure S12. $^{11}B\{^1H\}$ NMR spectra (Dimethoxyethane/C_6D_6) of BCTB in the presence of cyclohexene at room temperature, time zero (a), 80 °C (reaction temperature) after 23 h (b) and 80 °C (reaction temperature) after 72 h (c). Peaks related to BCTB, CTB, BF_3 (internal standard) and Cy_2BNH_2 are labelled with ●, ■, ▲ and ♦, respectively.

Catalytic decomposition of BCTB

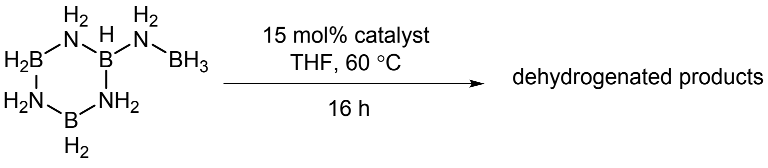

BCTB

In a nitrogen-filled glove box the catalyst precursor (15 mol% metal with respect to BCTB) was dissolved in THF (1.5 mL). To this stirred solution BCTB (from Zr-reaction, 20 mg in 1 mL THF) was added at room temperature and the resulting reaction mixture was stirred in a preheated bath (60 °C). NMR samples were taken after 1, 3 and 16 h. In the case of Ni-NHC and [Ru(*P^N*)Cl_2] the metal precursors were treated with the NHC ligand or KO(*t*-Bu) (1.0 equiv. with respect to Ru) at room temperature for several minutes prior to the addition of BCTB.

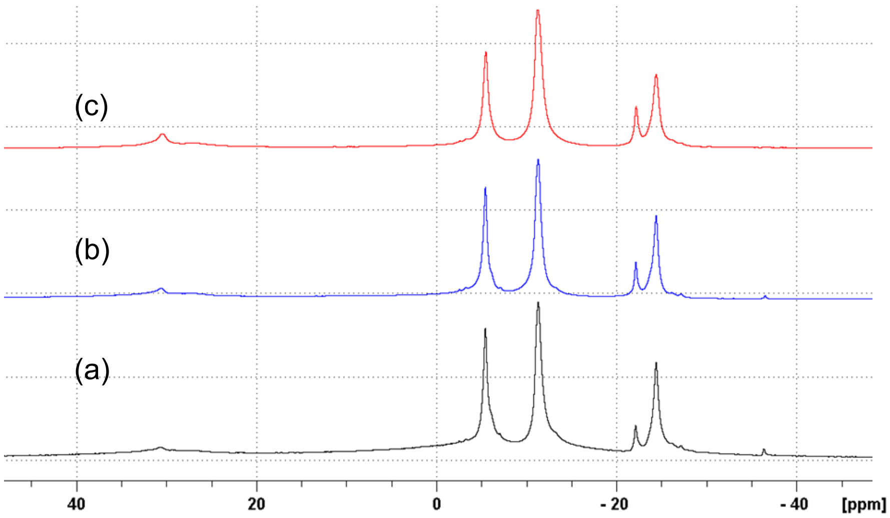

Figure S13. $^{11}B\{^{1}H\}$ NMR spectra (THF/C_6D_6) after thermal treatment (60 °C) of BCTB without catalyst for 1 (a), 3 (b), and 16 h (c).

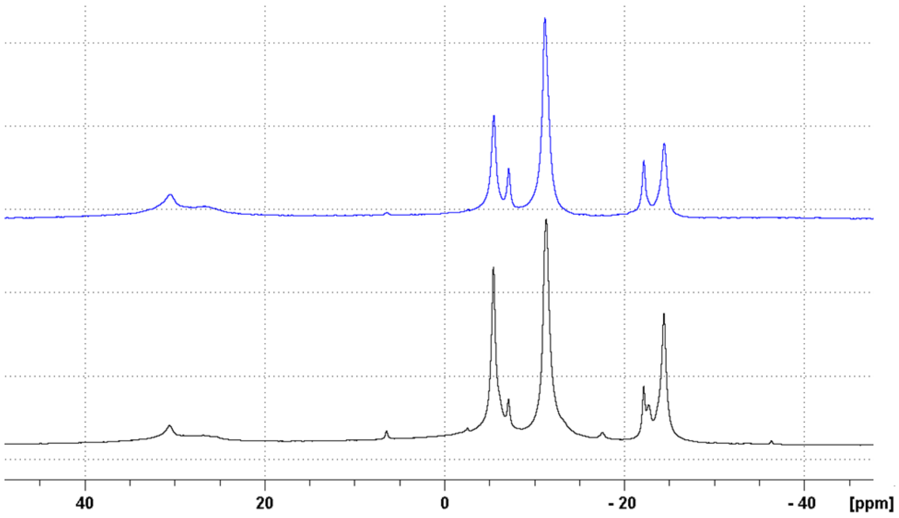

Figure S14. $^{11}B\{^{1}H\}$ NMR spectra (THF/C_6D_6) from 60 °C reaction of BCTB + 15 mol% [$(Cp)_2$ZrHCl] after 1 (bottom) and 16 h (top).

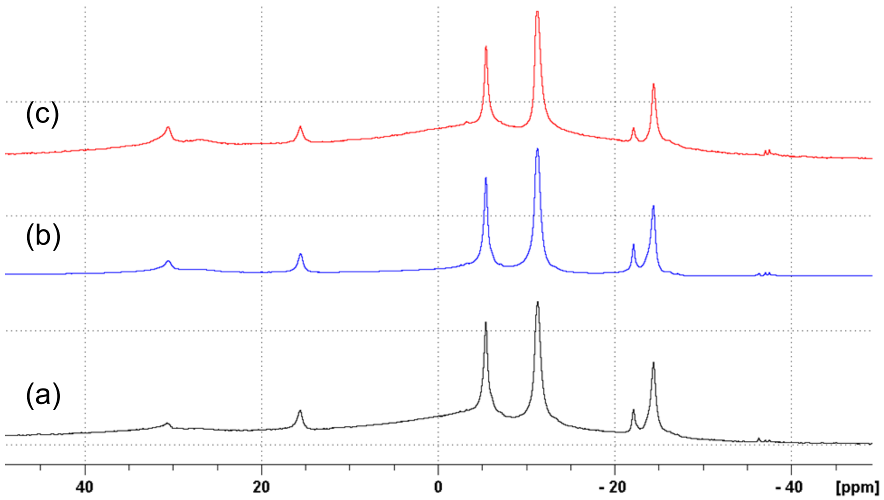

Figure S15. $^{11}B\{^1H\}$ NMR spectra (THF/C_6D_6) from 60 °C reaction of BCTB + 15 mol% [RuH(PMe_3)$_3$(BH_4)] after 1 (a), 3 (b), and 16 h (c).

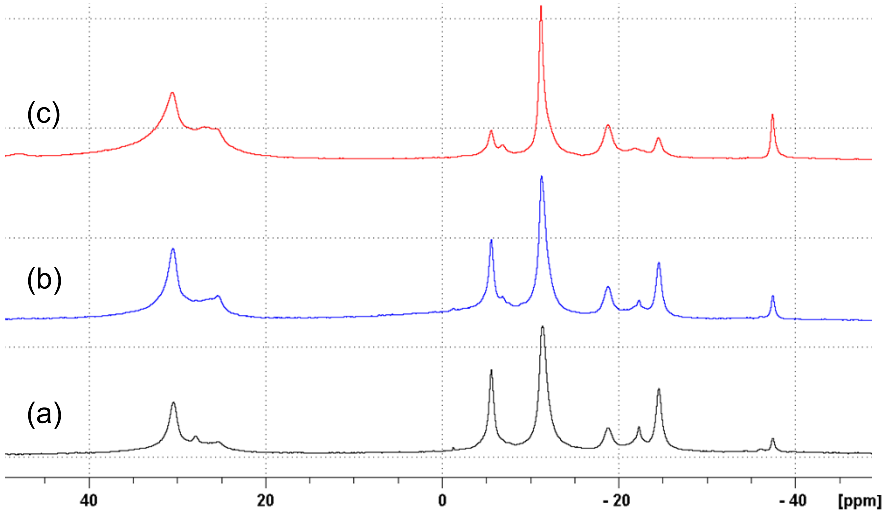

Figure S16. $^{11}B\{^1H\}$ NMR spectra (THF/C_6D_6) from 60 °C reaction of BCTB + 15 mol% [Ni(COD)$_2$] and 2 equiv. of NHC-ligand (NHC: 1,3-Bis(2,4,6-trimethylphenyl)-4,5-dihydroimidazol-2-ylidene) after 1 (a), 3 (b) and 16 h (c).

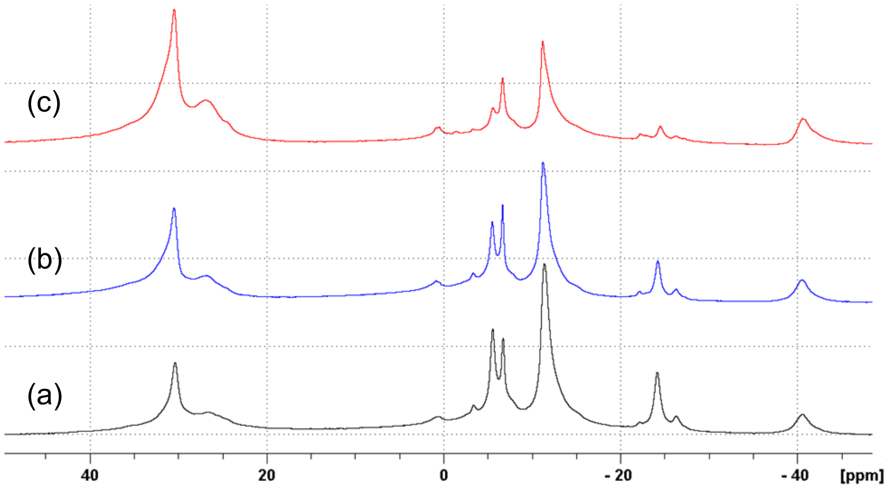

Figure S17. $^{11}B\{^{1}H\}$ NMR spectra (THF/C_6D_6) from 60 °C reaction of BCTB + 15 mol% [Fe(PP_3)H][BPh_4] (PP_3: Tris[2-(diphenylphosphino)ethyl]phosphine) after 1 (a), 3 (b) and 16 h (c).

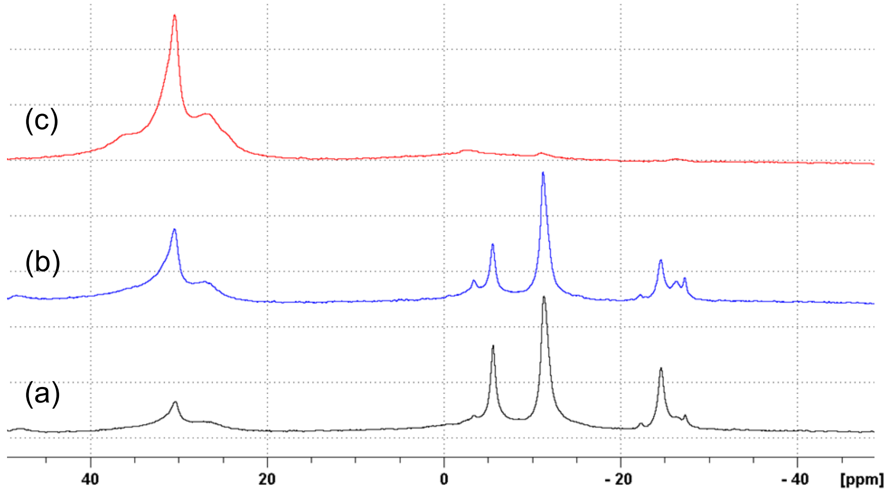

Figure S18. $^{11}B\{^{1}H\}$ NMR spectra (THF/C_6D_6) from 60 °C reaction of BCTB + 7.5 mol% [Rh(COD)Cl]$_2$ after 1 (a), 3 (b) and 16 h (c).

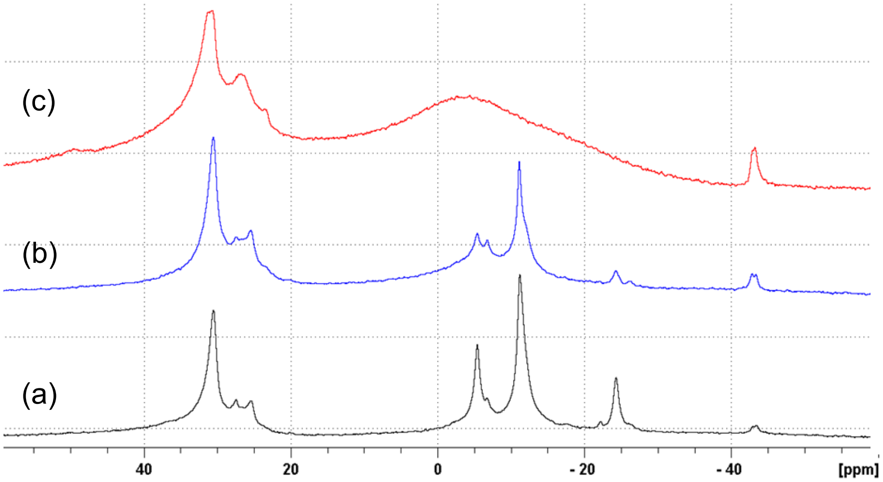

Figure S19. $^{11}B\{^1H\}$ NMR spectra (THF/C_6D_6) of 60 °C reaction of BCTB + 15 mol% [Ru(*N*^*P*)$_2$Cl$_2$] / KO(*t*-Bu) (*N*^*P*: 2-(di-t-butylphosphino)ethanamine)) after 1 (a), 3 (b) and 16 h (c).

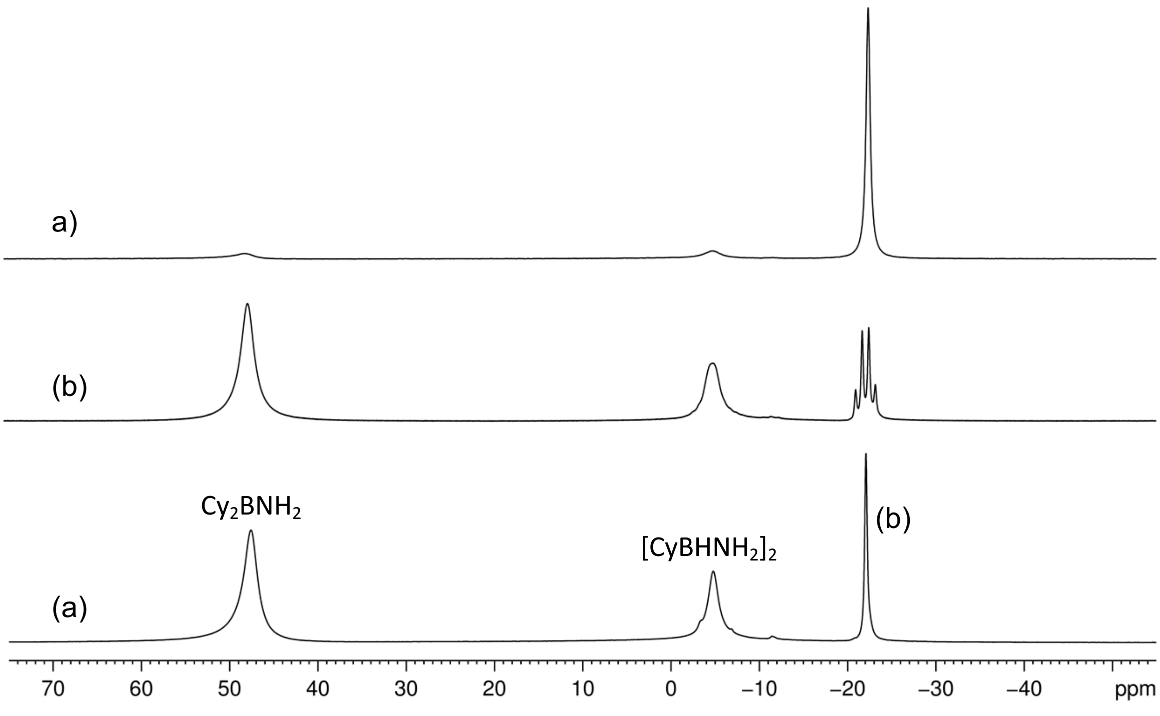

Figure S20. $^{11}B\{^1H\}$ (a and c) and ^{11}B (b) NMR spectra (THF/C_6D_6) of the reaction mixture from AB + cyclohexene (8 equiv.) in the presence of 5 mol% [Cp_2ZrHCl] (a and b) and control reaction (c) at 50 °C for 24 h.

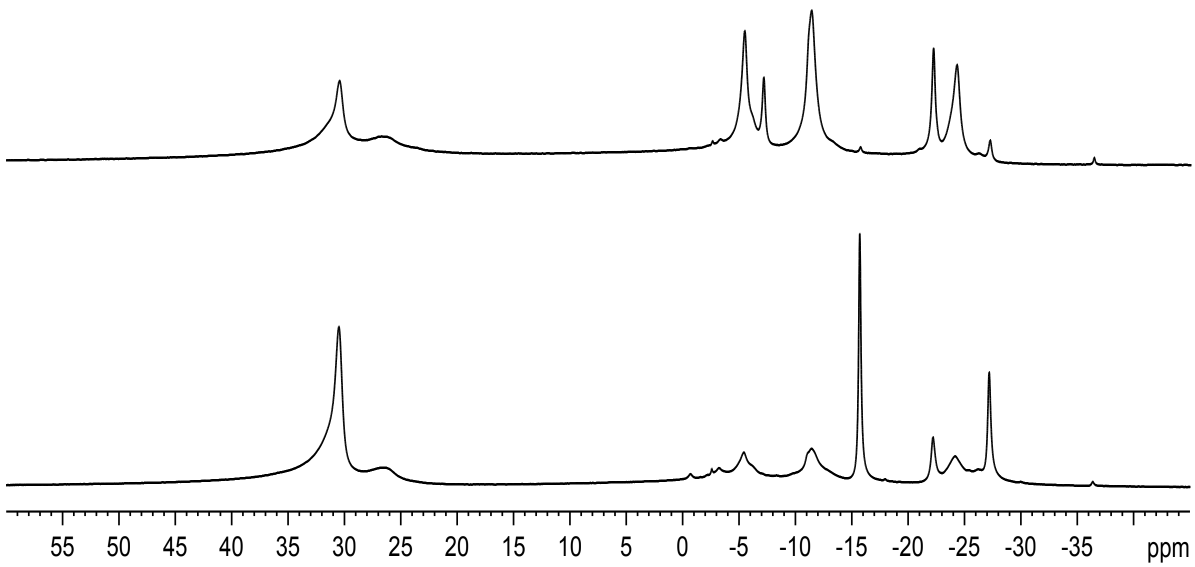

Figure S21. Comparison of the $^{11}B\{^{1}H\}$ NMR spectra (THF/C_6D_6) of the reaction mixture from AB + 5 mol% [Cp_2ZrHCl] (top) and $Cp_2Zr(BH_4)_2$ (bottom) at 50 °C after 48 h.

References

[1] APEX Software Suite v.2010; Bruker AXS: Madison, WI, **2005**.

[2] R. Blessing, *Acta Cryst.* 1995, **A51**, 33.

[3] G. M. Sheldrick, *Acta Cryst.* 2008, **A64**, 112.

[4] H. H. Karsch, H-F. Klein, H. Schmidbauer, *Chem. Ber*. 1977, **110**, 2200-2212.
